# d-Amino Acid Peptide Residualizing Agents for Protein Radioiodination: Effect of Aspartate for Glutamate Substitution

**DOI:** 10.3390/molecules23051223

**Published:** 2018-05-20

**Authors:** Marek Pruszynski, Choong Mo Kang, Eftychia Koumarianou, Ganesan Vaidyanathan, Michael R. Zalutsky

**Affiliations:** 1 Department of Radiology, Duke University Medical Center, Durham, NC 27710, USA; kcm1580@naver.com (C.M.K.); eftychia.koumarianou@duke-nus.edu.sg (E.K.); ganesan.v@duke.edu (G.V.); 2 Present address: Institute of Nuclear Chemistry and Technology, 03-195 Warsaw, Poland; 3 Present address: Korea Institute of Radiological and Medical Sciences, Seoul 01812, Korea; 4 Present address: Laboratory for Translational and Molecular Imaging, Duke-National University of Singapore Medical School, Singapore 169857, Singapore

**Keywords:** radioiodination, VHH, HER2, residualizing label, breast cancer

## Abstract

The residualizing prosthetic agent *N*^ε^-(3-[^*^I]iodobenzoyl)-Lys^5^-*N*^α^-maleimido-Gly^1^-d-GEEEK ([^*^I]IB-Mal-d-GEEEK) showed promise for the radioiodination of monoclonal antibodies (mAbs) that bind to internalizing molecular targets. Although enhanced tumor uptake was achieved in these studies, elevated kidney accumulation also was observed, particularly with low-molecular-weight, single-domain antibody fragments (sdAbs). Here, we developed an analogous agent (IB-Mal-d-GDDDK), in which glutamate residues (E) were replaced with aspartates (D) to determine whether this modification could decrease renal uptake. [^125^I]IB-Mal-d-GDDDK and [^131^I]IB-Mal-d-GEEEK were synthesized with similar radiochemical yields (60–80%) and coupled to the anti-HER2 sdAb 5F7 at 50–60% efficiency. Paired-label internalization assays in vitro indicated similar levels of intracellular activity residualization in HER2-expressing BT474M1 cells for [^125^I]IB-Mal-d-GDDDK-5F7 and [^131^I]IB-Mal-d-GEEEK-5F7. A paired-label biodistribution comparison of the two labeled conjugates was performed in mice with HER2-expressing SKOV-3 xenografts, and the results of this study indicated that renal uptake at 1 h was 127.5 ± 18.7% ID/g and 271.4 ± 66.6% ID/g for [^125^I]IB-Mal-d-GDDDK-5F7 and [^131^I]IB-Mal-d-GEEEK-5F7, respectively. The tumor uptake of the two radioconjugates was not significantly different. These results demonstrate that substitution of E with D in the IB-Mal-d-GEEEK construct reduced kidney accumulation of the sdAb. However, renal activity levels need to be reduced further if d-amino acid derived prosthetic agents are to be of practical value for labeling low molecular weight biomolecules such as sdAbs.

## 1. Introduction

Interest in radiolabeled proteins and peptides as clinical diagnostics and therapeutics has been increasing during the last decade [1,2]. However, each type of radionuclide requires a different labeling method, which can have a significant influence on the biodistribution of the labeled tracer [3]. Differences in biodistribution have been observed even for radionuclides belonging to the same periodic group [4,5,6]. Therefore, matched pair radiometals such as ^64^Cu/^67^Cu, ^43/44^Sc/^47^Sc, and ^86^Y/^90^Y are well-suited for theranostic applications. For theranostics, iodine radioisotopes represent an attractive alternative to radiometals because of the ready availability of radioisotopes for SPECT (^123^I) or PET (^124^I) imaging, as well as β^−^-particle (^131^I) or Auger electron (^123^I, ^125^I) radiotherapy, at a reasonable cost.

Unfortunately, direct electrophilic radioiodination of tyrosine residues in proteins, while simple, is not a suitable method for labeling internalizing biomolecules, because the lysosomal degradation products of such labeled proteins, monoiodotyrosine and free iodide, are rapidly washed out from tumor cells and can accumulate in the thyroid and stomach [7]. This problem has been overcome through the use of residualizing agents, which generally contain charged or polar moieties that can be trapped within the lysosomes after biomolecule internalization and degradation. A variety of residualizing radioiodination agents have been developed by our group and by others [8,9,10,11,12,13]. One of the most promising of these is *N*^ε^-(3-[^*^I]iodobenzoyl)-Lys^5^-*N*^α^-maleimido-Gly^1^-d-GEEEK ([^*^I]IB-Mal-d-GEEEK), which consists of a pentapeptide that includes three negatively charged d-glutamic acids to facilitate intracellular trapping, a deiodination-resistant iodobenzoyl group ([^*^I]IB) attached to a d-lysine side chain, and a maleimide (Mal) functionality for conjugation to sulfhydryl groups on a biomolecule [14]. This prosthetic group offered clear advantages as a reagent for labeling internalizing intact mAbs and larger mAb fragments, including excellent retention of activity in tumor xenografts, minimal dehalogenation, and high tumor-to-normal tissue ratios [14,15]. Based on these results, [^*^I]IB-Mal-d-GEEEK was evaluated for the radioiodination of a rapidly internalizing single domain antibody fragment (sdAb), a protein format derived from Camelid heavy-chain-only antibodies that is also known as VHH molecules or nanobodies. Among their favorable properties, sdAbs have a size (~15 kDa) of about a tenth of intact mAbs, which facilitates their rapid tumor targeting and clearance from background tissues [16]. A promising example of the sdAb class of targeting agents is 5F7, which targets the human epidermal growth factor receptor type 2 (HER2) [17,18]. Use of [^*^I]IB-Mal-d-GEEEK to label 5F7 sdAb resulted in favorable accumulation in HER2-expressing BT474M1 xenografts. However, very high and prolonged kidney uptake (>100% ID/g) also was observed, which would be problematic, particularly if therapeutic application were envisioned.

With regard to the use of d-amino acid peptides as residualizing prosthetic agents for radioiodination, our initial strategy was to utilize a peptide containing three positively charged d-arginines [8]. While excellent tumor accumulation was achieved with an internalizing mAb radioiodinated using this residualizing prosthetic agent, very high kidney uptake also was seen. Because positively charged molecules can bind to negatively charged renal proximal tubules [19], it was thought that the observed high renal accumulation might reflect an interaction of this type. To potentially overcome this drawback, [^*^I]IB-Mal-d-GEEEK was developed with the rationale that negatively charged, labeled catabolites would not bind to renal proximal tubules [20]. Unfortunately, high kidney retention remained a problem when smaller size sdAbs were labeled using [^*^I]IB-Mal-d-GEEEK [17,18].

In studies evaluating ^11^C-labeled amino acids as tracers for neuroendocrine tumor imaging, Wu et al. [21] observed that the renal accumulation of [^11^C]aspartate in rats and humans was significantly lower than that for [^11^C]glutamate. This provided motivation for the current study investigating whether replacing d-glutamate (E) in the [^*^I]IB-Mal-d-GEEEK template with d-aspartate (D) would reduce kidney accumulation of activity from radioiodinated internalizing proteins without affecting tumor uptake. A 5F7 sdAb was chosen for this comparison, because kidney activity is much more of an issue for sdAbs than mAbs [17,18,22]. In this study, [^*^I]IB-Mal-d-GDDDK was synthesized, and 5F7 sdAb was radiolabeled using this prosthetic agent. The resultant radiolabeled sdAb conjugate was directly compared with that labeled using [^*^I]IB-Mal-d-GEEEK in vitro in HER2-expressing BT474M1 human breast carcinoma cells and in vivo in athymic mice bearing HER2-expressing SKOV-3 human ovarian carcinoma xenografts.

## 2. Results and Discussion

Due to the small mass of sdAbs, they clear rapidly from blood and normal tissues and can easily penetrate tumors [16]. These molecules have delivered excellent results in high-contrast imaging of tumor-associated biomarkers both in preclinical [23,24,25] and in a pilot clinical proof-of-principle study [26]. The 5F7 sdAb is an attractive targeting agent for cancer diagnosis and therapy due to its nanomolar affinity for HER2 and rapid internalization after receptor binding [17]. Because it internalizes extensively, a residualizing radiolabeled prosthetic agent that minimizes loss of activity from tumor cells after receptor-mediated endocytosis and lysosomal degradation is needed to maximize its potential utility for radioimaging and targeted radiotherapy. Although [^*^I]IB-Mal-d-GEEEK offered significant advantages over conventional methods for radioiodination of 5F7 sdAb with respect to tumor uptake, unfortunately, its use also resulted in more than an order of magnitude higher kidney accumulation compared with 5F7 sdAb radioiodinated with Iodogen, with kidney uptake greater than 100% ID/g at all-time points studied [17,18]. It should be pointed out that high kidney uptake also has been observed with other radiolabeled molecules containing glutamic acid [27], and attempts have been made to reduce their renal activity by coadministering polyglutamic acid [28]. Although coinjection of Gelofusine or a cocktail of amino acids might ameliorate kidney uptake [29], a more direct and clinically amenable approach would be to modify the structure of the [^*^I]IB-Mal-d-GEEEK reagent to address the renal accumulation issue. Hypothesizing that high kidney uptake of activity from 5F7 radioiodinated using [^*^I]IB-Mal-d-GEEEK might be due to the glutamate residues in the prosthetic agent, we assumed that replacing these glutamates with aspartates (yielding [^*^I]IB-Mal-d-GDDDK) would reduce kidney uptake without compromising tumor uptake.

To assess the potential effect of substituting aspartic acid for glutamic acid on kidney uptake, an initial study was performed to compare the biodistribution of two d-amino acid pentapeptides in normal mice. In both peptides, a tyrosine was included to facilitate radioiodination. In one peptide, there were four glutamates, in addition to tyrosine (d-YEEEE), while the other peptide contained four aspartates (d-YDDDD). As shown in Table S1, d-[^125^I]YDDDD and d-[^131^I]YEEEE exhibited markedly different radioiodine distribution patterns. With the exception of the intestines, tissue activity levels generally were significantly higher for d-[^125^I]YDDDD. For example, ^125^I activity levels in the liver, spleen, and lungs was almost 30% higher than those for ^131^I at 30 min and about 2–3-times higher at 24 h. On the other hand, kidney levels of d-[^125^I]YDDDD were 3–9-fold lower compared with those seen for d-[^131^I]YEEEE over the 24-h experimental period. These promising results motivated us to synthesize [^*^I]IB-Mal-d-GDDDK and evaluate its potential utility for labeling the rapidly internalizing anti-HER2 sdAb, 5F7.

**Scheme 1:**
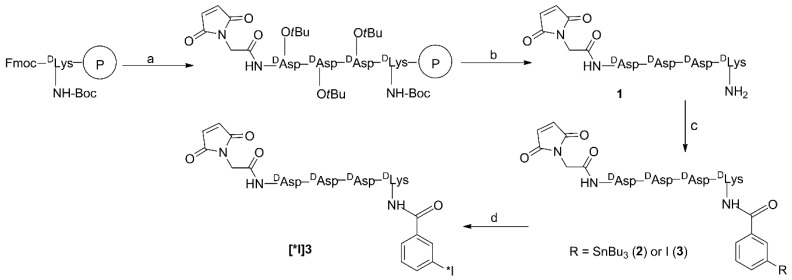
Syntheses of tin precursor **2**, IB-Mal-d-GDDDK, and [*I]IB-Mal-d-GDDDK: (a) solid-phase peptide synthesis (SPPS): addition of three d-Asp-(O*t*Bu)-OH residues and one maleimido glycine (maleimido acetic acid); (b) 100:95:2.5:2.5 DCM:TFA:H_2_O:TIS cleavage cocktail; (c) *N*-succinimidyl 3-(tri-*n*-butyl)stannyl-benzoate (for **2**) or *N*-succinimidyl 3-iodobenzoate (for **3**), TEA/DMF; (d) **2**, radioiodine, NCS, acetic acid.

Nonradioactive IB-Mal-d-GDDDK and its corresponding tin precursor were synthesized as depicted in Scheme 1. The pentapeptide Mal-d-GDDDK (**1**), obtained by solid-phase peptide synthesis and subsequent cleavage from the resin, was conjugated with *N*-succinimidyl 3-(tri-*n*-butyl)stannyl-benzoate (STB) and *N*-succinimidyl 3-iodobenzoate (SIB) to render the tin precursor **2** and nonradioactive iodo-standard **3**, respectively, in moderate yields. The molecular weights found by mass spectrometry for Mal-d-GDDDK, as well as its STB and SIB conjugates, were consistent with theoretically calculated values.

**Figure 1:**
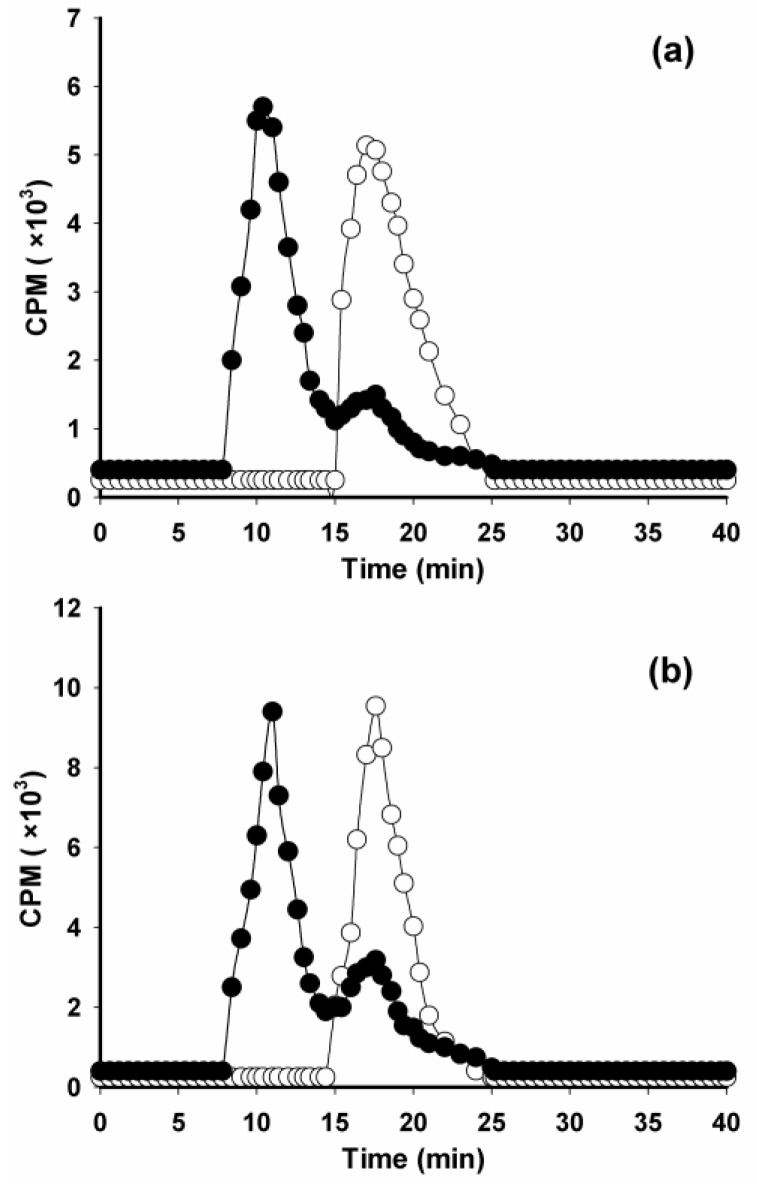
Size-exclusion HPLC chromatography of radioiodinated 5F7 sdAb (open circles) and after incubation with 10–20-fold excess of HER2-ECD at 37 °C for 30 min (filled circles): (**a**) [^125^I]IB-Mal-d-GDDDK-5F7; (**b**) [^125^I]IB-Mal-d-GEEEK-5F7.

**Figure 2:**
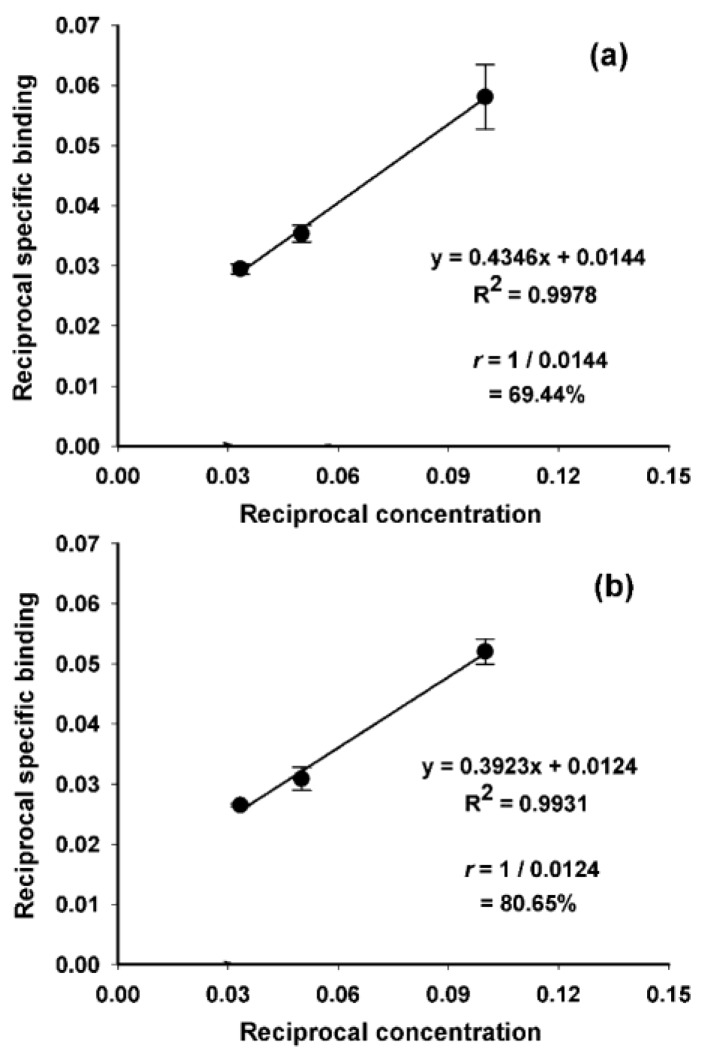
Representative Lindmo immunoreactivity assay data: (**a**) [^125^I]IB-Mal-d-GDDDK-5F7; (**b**) [^125^I]IB-Mal-d-GEEEK-5F7.

The tin precursor **2** was radioiodinated to obtain [^125^I]IB-Mal-d-GDDDK ([^125^I]**3**; Scheme 1) in 67.4 ± 22.2% (*n* = 10) yield and with a 97.4 ± 1.5% radiochemical purity after isolation by reversed-phase HPLC. The coupling efficiency of [^125^I]IB-Mal-d-GDDDK to 5F7 was 57.0 ± 9.5% (*n* = 9). These results are similar to those obtained for [^131^I]IB-Mal-d-GEEEK in the current study: radioiodination yields of 76.5 ± 10.9% (*n* = 6), a radiochemical purity of 97.5 ± 1.1%, and 5F7 conjugation yields of 55.4 ± 10.1% (*n* = 6). The conjugation efficiency for [^125^I]IB-Mal-d-GDDDK also was comparable to the results obtained previously for the coupling of [^*^I]IB-Mal-d-GEEEK to larger proteins [14,15,22], sdAbs [17,18], and those reported for other maleimide-containing agents used for radiolabeling biomolecules such as affibodies [30]. The molar activity for [^125^I]IB-Mal-d-GDDDK-5F7 and [^131^I]IB-Mal-d-GEEEK-5F7 was in the range of 0.4–7.1 MBq/nmol and 2.6–6.4 MBq/nmol, respectively, which largely reflected the activity level of the prosthetic agent used for conjugation. Higher molar activities, if needed, are likely obtainable by starting with higher amounts of activity. ITLC and TCA-precipitation assays indicated that 98.3 ± 1.1% and 98.6 ± 0.9% of the activity was protein-associated for both radioconjugates, respectively. The SDS-PAGE revealed that 97.2 ± 2.2% of activity was protein-associated (Figure S1). When analyzed by size-exclusion HPLC, [^125^I]IB-Mal-d-GDDDK-5F7 and [^131^I]IB-Mal-d-GEEEK-5F7 showed a single major peak with a retention time of 17.2 min, consistent with a molecular weight of about 15 kDa (Figure 1). When the labeled sdAbs were incubated with an excess of HER2-ECD, 70–80% of the activity shifted to a retention time of 10.3 min, corresponding to a molecular weight of about 145 kDa, indicating binding of the radioconjugates to HER2-ECD (Figure 1). The immunoreactive fractions, determined using a modified Lindmo assay, were 63.1 ± 10.8% and 70.5 ± 10.0% (*n* = 3) for [^125^I]IB-Mal-d-GDDDK-5F7 and [^131^I]IB-Mal-d-GEEEK-5F7, respectively (Figure 2). These values are comparable to those obtained when 5F7 sdAb was labeled using other radioiodination methods [13,17,18,31], suggesting that its immunoreactivity was not compromised by modification with the novel prosthetic agent, [^*^I]IB-Mal-d-GDDDK.

**Figure 3:**
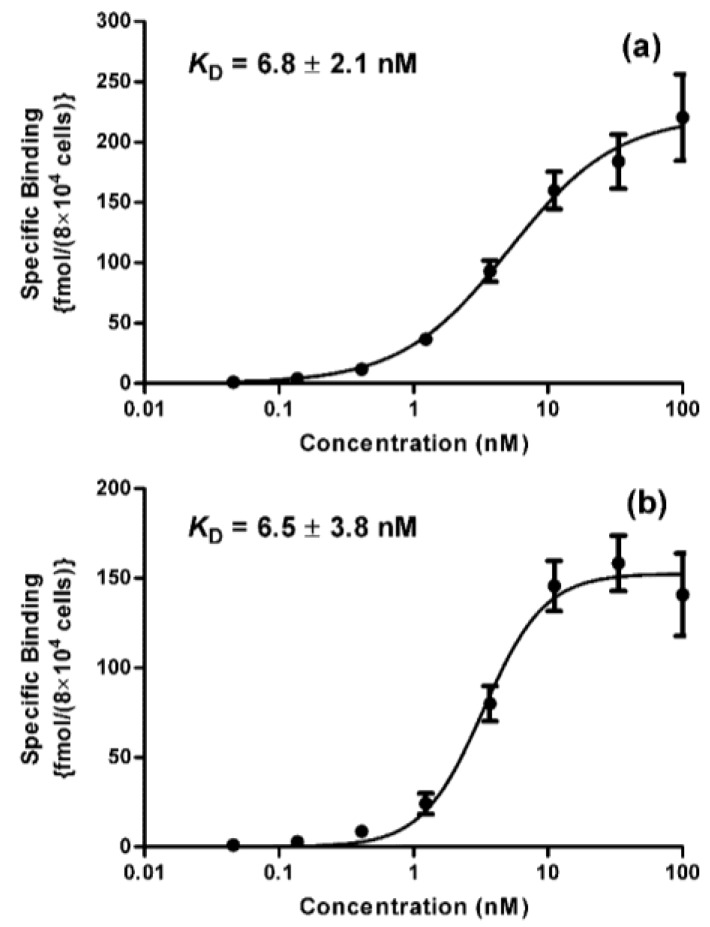
Saturation binding assay data obtained using BT474M1 cells: (**a**) [^125^I]IB-Mal-d-GDDDK-5F7; (**b**) [^125^I]IB-Mal-d-GEEEK-5F7. Data presented as average ± SD of two independent experiments performed in triplicates for each concentration of the radioconjugates.

The binding affinity of [^125^I]IB-Mal-d-GDDDK-5F7 and [^131^I]IB-Mal-d-GEEEK-5F7 was evaluated using the HER2-expressing BT474M1 human breast carcinoma cells. The calculated percentage of non-specific binding during coincubation with a 100-fold molar excess of the anti-HER2 antibody trastuzumab was in the range of 2–20% with increasing radioconjugates concentration (0.1–100 nM). The equilibrium dissociation constant (*K*_D_) values calculated for [^125^I]IB-Mal-d-GDDDK-5F7 and [^125^I]IB-Mal-d-GEEEK-5F7 were 6.8 ± 2.1 nM and 6.5 ± 3.8 nM, respectively (Figure 3); these values are similar to those reported, also using BT474M1 cells, for 5F7 sdAb labeled with ^125/131^I, ^18^F, or ^211^At [17,18,31,32]. These values are also similar to those obtained on BT474M1 cells when trastuzumab was labeled using [^125^I]IB-Mal-d-GEEEK (5.4 ± 0.7 nM) or a similar prosthetic agent [^125^I]IB-NHS-d-EEEG (5.7 ± 0.6 nM) [22].

**Figure 4:**
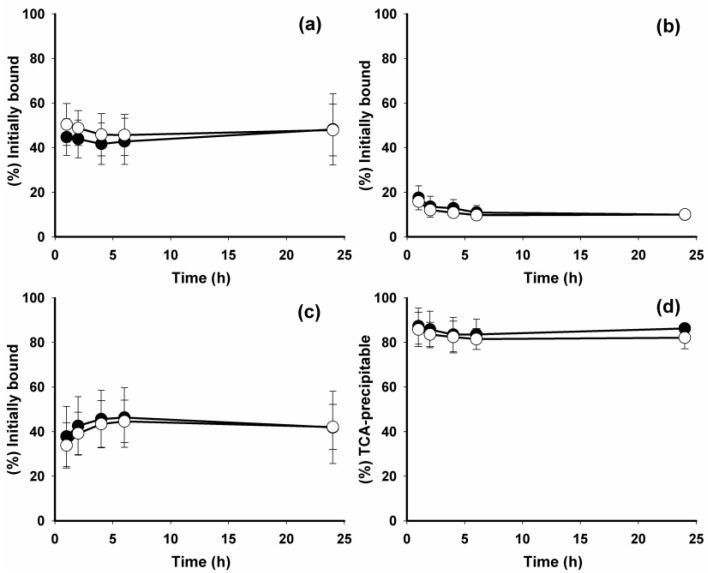
Paired-label internalization of [^125^I]IB-Mal-d-GDDDK-5F7 (filled circles) and [^131^I]IB-Mal-d-GEEEK-5F7 (open circles) by BT474M1 cells. Data presented as (**a**) the percentage of initially bound activity (specific) that was internalized, (**b**) membrane-bound, (**c**) in the cell culture supernatants, and (**d**) activity in the cell culture supernatants that was TCA-precipitable.

[^*^I]IB-Mal-d-GDDDK, analogous to [^*^I]IB-Mal-d-GEEEK, was designed to be a residualizing agent based on the hypothesis that a proteolytically inert d-peptide with negatively charged amino acids would not be transported across lysosomal and cell membranes. To investigate whether exchanging glutamate for aspartate affected residualizing capability, a paired-label assay was performed to directly compare the internalization and cell processing of [^125^I]IB-Mal-d-GDDDK-5F7 and [^131^I]IB-Mal-d-GEEEK-5F7 on BT474M1 cells (Figure 4). The cellular uptake of both radioconjugates was demonstrated to be HER2-mediated by the fact that coincubation with a 100-fold molar excess of trastuzumab reduced uptake and internalization to less than 0.3% of control values. As shown in Figure 4a, the percentage of initially bound activity that was in the intracellular compartment was quite similar and fairly constant for both labeled 5F7 sdAb conjugates throughout the 24-h observation period. For example, 44.7 ± 8.2% of initially bound [^125^I]IB-Mal-d-GDDDK-5F7 and 50.4 ± 9.4% of [^131^I]IB-Mal-d-GEEEK-5F7 activity was internalized at 1 h; at 24 h, these values were 48.2 ± 16.0% and 47.9 ± 11.6%, respectively. Although [^131^I]IB-Mal-d-GEEEK-5F7 showed a slightly higher intracellular trapping compared with [^125^I]IB-Mal-d-GDDDK-5F7 at early time-points, the difference was not statistically significant (*p* > 0.05). These results are in good agreement with those obtained previously with [^*^I]IB-Mal-d-GEEEK-5F7 [17,18]. These data indicate that substitution of glutamates with aspartates did not compromise residualizing capability in the new prosthetic agent. Membrane-bound activity (Figure 4b) was similarly low for both tracers at all-time points. The amount of activity released into the cell culture supernatants (Figure 4c) was complementary to the results observed for intracellular trapping. The slight increase of activity present in the cell culture medium with time could be due to dissociation of the sdAb from its cell-surface receptor, as a similar amount of activity was lost from membrane during the 24-h incubation. Several other processes including receptor recycling and/or generation of labeled catabolites also may play a role. To investigate the latter, the TCA precipitability of activity present in the cell culture supernatants was determined. The fraction of activity that remained protein-associated was high and fairly constant for both 5F7 radioconjugates throughout the 24-h experiment (Figure 4d). For example, the fraction of activity in the cell culture supernatants that was protein-associated was 87.3 ± 8.1% for [^125^I]IB-Mal-d-GDDDK-5F7 and 85.8 ± 7.7% for [^131^I]IB-Mal-d-GEEEK-5F7 at 1 h; these values were 86.3 ± 2.2% and 82.1 ± 5.0%, respectively, at 24 h. These results indicate a very low rate of escape of low molecular weight catabolites from the cells for both 5F7 sdAb conjugates.

**Figure 5:**
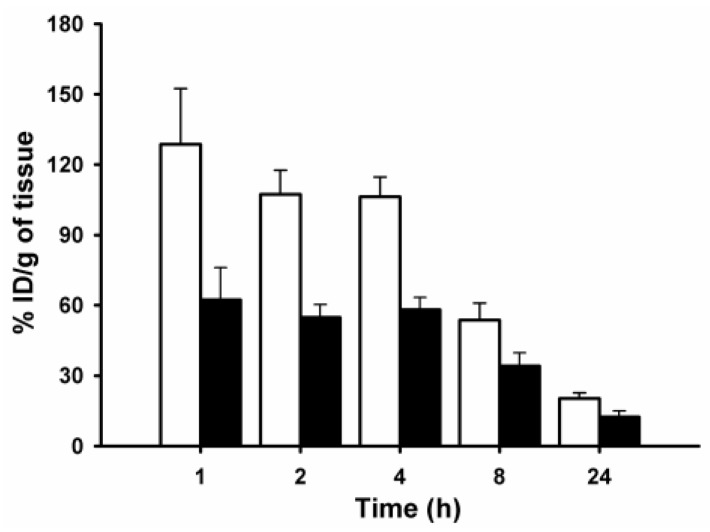
Paired-label kidney uptake of [^125^I]IB-Mal-d-GDDDK-5F7 (closed bars) and [^131^I]IB-Mal-d-GEEEK-5F7 (open bars) in Balb/c mice.

We next evaluated the biodistribution of 5F7 sdAb radioiodinated using [^125^I]IB-Mal-d-GDDDK in comparison with [^131^I]IB-Mal-d-GEEEK-5F7 in Balb/c mice. The results of this study are given in Table S2. Consistent with the results obtained with the two pentapeptides described above, the most significant difference (*p* < 0.0002) observed in the biodistribution of the sdAb radioconjugates was the almost 2-fold lower kidney uptake of [^125^I]IB-Mal-d-GDDDK-5F7 compared with [^131^I]IB-Mal-d-GEEEK-5F7 over 24-h study (Figure 5). Based on these encouraging results, we evaluated the tissue distribution of the two labeled 5F7 conjugates in athymic mice with subcutaneous HER2-expressing SKOV-3 ovarian carcinoma xenografts (Table 1). The general biodistribution pattern of both radioconjugates was typical for sdAb molecules, with a rapid clearance from blood and other normal tissues observed. However, compared with [^131^I]IB-Mal-d-GEEEK-5F7, [^125^I]IB-Mal-d-GDDDK-5F7 exhibited a higher accumulation in lungs, liver, and spleen, with an approximately 2-fold higher uptake observed at 1 h, with the difference increasing to 4-fold at 24 h. It is worth noting that the aspartate-containing pentapeptide described above also showed higher uptake relative to its glutamate analogue in these tissues in normal mice. The higher accumulation of [^125^I]IB-Mal-d-GDDDK-5F7 in these organs might be due to a number of factors, including deiodination in vivo, because elevated uptake of free iodide in these organs has been reported [7]. However, differential deiodination is unlikely, because both templates have the same iodobenzoyl ([^*^I]IB) moiety. Moreover, radioiodine levels in stomach and thyroid (% ID/organ), tissues known to take up iodide avidly [7] were very low, suggesting a similar low degree of in vivo deiodination for both radioconjugates.

With regard to differences in the normal tissue distribution pattern of the two 5F7 sdAb conjugates, previous preclinical and clinical studies with ^68^Ga-labeled 2Rs15d, another HER2-targeted sdAb, revealed that the mass of protein administered had a significant effect on accumulation levels in normal organs such as the liver, spleen, and lungs [23,26]. For example, the uptake of ^68^Ga-labeled 2Rs15d in lungs and liver at 1 h was 8.3 ± 4.9% ID/g and 7.4 ± 1.9% ID/g, respectively, at a 0.1 μg dose and 0.5 ± 0.1% ID/g and 2.9 ± 0.3% ID/g, at a 10 μg dose [23]. However, the higher uptake of [^125^I]IB-Mal-d-GDDDK-5F7 compared with [^131^I]IB-Mal-d-GEEEK-5F7 in these organs cannot be explained by injected mass, because this was a paired-label study, and the total amount of sdAb administered should have the same effect on both radioconjugates. Thus, the higher accumulation of [^125^I]IB-Mal-d-GDDDK-5F7 in lungs, spleen, and liver likely reflects the substitution of glutamate for aspartate in the prosthetic agent, which is corroborated by the differences observed in the biodistribution of d-[^125^I]YDDDD and d-[^131^I]YEEEE (see above). Consistent with these results, it has been reported that the uptake of [^14^C]aspartate in rat liver, spleen, and lungs was 2–3-fold higher than that for [^14^C]glutamate as early as 10 min after administration [33]. Similarly, higher uptake of [^11^C]aspartate compared to [^11^C]glutamate in rat liver and human spleen was observed in a study evaluating these amino acids as potential tracers for PET imaging of neuroendocrine tumors [21]. However, the reason for the differential uptake of these two amino acids in these organs is not clear.

The most notable finding from our biodistribution study is the much lower renal uptake of [^125^I]IB-Mal-d-GDDDK-5F7 compared to that for [^131^I]IB-Mal-d-GEEEK-5F7 without a concomitant difference in tumor uptake (Table 1). Tumor accumulation of the two radioconjugates over a 24-h period was comparable (*p* > 0.05) and was similar to that seen for [^*^I]IB-Mal-d-GEEEK-5F7 in a previous study, albeit in a different HER2-expressing xenograft model (BT474M1) [17,18]. In the current study, peak tumor uptake occurred at 1 h with both radioconjugates at levels (4–5% ID/g) that were similar to those (2.5–6.0% ID/g) reported in the same SKOV-3 model for 2Rs15d (3–4 μg), another anti-HER2 sdAb radiolabeled with ^68^Ga, ^131^I and ^225^Ac [23,34,35]. On the other hand, tumor uptake observed in the current study in terms of both peak levels and fraction retained at later time points were less favorable than those observed in the BT474M1 xenograft model for 5F7 labeled with [*I]SGMIB or its *iso*-analogue [18,32]. However, comparisons of results obtained in different xenograft models must be done with caution because of differences that might exist in HER2 receptor expression, tumor hemodynamics, and other factors that might affect tumor localization. For example, in a recent study evaluating the tissue distribution of [^*^I]SGMIB-2Rs15d sdAb conjugates, the tumor uptake observed in BT474M1 xenografts was 2–3 times higher than that were seen in SKOV-3 xenografts [34].

In tumor-bearing mice, kidney uptake of [^125^I]IB-Mal-d-GDDDK-5F7 was almost 2-fold lower than that for [^131^I]IB-Mal-d-GEEEK-5F7 at 1 and 4 h; however, no differences were observed at 24 h (Table 1). This was somewhat surprising, because in non-tumor bearing Balb/c mice, lower kidney uptake of [^125^I]IB-Mal-d-GDDDK compared to [^131^I]IB-Mal-d-GEEEK also was seen (*p* < 0.0001) at 24 h (Table S2). Although the reason for the difference between the two experiments is not clear, direct comparison of biodistribution results performed in different mouse strains is not straightforward. The intrinsic properties of a biomolecule, among other factors, dictate the kidney retention of activity after radiolabeling. Very high kidney uptake has been observed for radiolabeled peptides [29], affibodies [36], and sdAbs [17,37], with levels as high as 250% ID/g in murine studies. Molecules with a molecular weight below 60 kDa are accumulated to a higher degree in the renal cortex, which is responsible for reabsorption of molecules filtered by the glomeruli. A major contributor to this phenomenon is the highly expressed endocytic megalin-receptor system. For example, it has been shown that the renal uptake of ^99m^Tc-7C12-EGFR sdAb was 235.8 ± 7.7% ID/g in megalin-expressing mice compared to 131.5 ± 8.2% ID/g in megalin-deficient mice [37].

With regard to understanding the renal uptake of labeled biomolecules, it is important to bear in mind that in addition to protein size, one must also consider effects related to the nature of the radiolabeling method. So-called residualizing labels can be trapped not only in the lysosomes of cancer cells, but also in cells in liver, spleen, and kidney involved in the metabolism of proteins [10]. [^*^I]IB-Mal-d-GEEEK and [^*^I]IB-Mal-d-GDDDK were designed to possess three negatively charged amino acids in order to impart high residualizing properties. This presumably accounts for the high and prolonged tumor accumulation observed when an intact mAb was labeled with this acylation agent [14]; however, this residualizing property also might have contributed to 10–25-fold higher renal uptake of [^131^I]IB-Mal-d-GEEEK-5F7 compared to directly iodinated 5F7 sdAb [17]. When 5F7 sdAb was labeled with another type of residualizing agent, [^*^I]SGMIB, activity levels in the kidneys, and, to a lesser degree, accumulation in liver and spleen, was considerably lower compared with [^*^I]IB-Mal-d-GEEEK-5F7 sdAb [18]. The difference in polarity of [^*^I]SGMIB and [^*^I]IB-Mal-d-GEEEK could have an influence on their residualization capability and, in turn, on renal uptake. With intact mAbs and other proteins above the size cut-off for renal filtration, such differences are less critical; however, for molecules like an sdAb, such differences become much more important.

In the current study, the observed differences in kidney accumulation of [^125^I]IB-Mal-d-GDDDK-5F7 and [^131^I]IB-Mal-d-GEEEK-5F7 might not be due to their differential residualization capacity. According to the results obtained with BT474M1 cells in vitro, internalization and intracellular trapping of radioiodine was similar with both tracers (Figure 4), which was expected given that the net charge of both prosthetic agents is the same. A possible explanation for higher kidney retention of activity from biomolecules labeled using [^*^I]IB-Mal-d-GEEEK is that its constituent glutamic acid residues might be recognized by the several anionic amino acid transporters in mammalian kidney, especially EAAT3, a major transporter for l-glutamic acid [15,38]. However, if glutamate retention in the kidney is mediated by EAAT3, it is likely that aspartate also could be recognized by the same transport mechanism. Indeed, it has been reported that EAAT3 can mediate the uptake of d-aspartate in SH-SY5Y neuroblastoma cells [39]. Also, Schuldt et al. [40] noted a transporter at the antiluminal surface of renal tubule cells (EAAC1) that can transport both d-aspartate and d-glutamate. However, as noted earlier, Koch et al. [33] reported that [^14^C]glutamate had 2-fold higher retention in rat kidneys than [^14^C]aspartate as early as 10 min after administration. Similar results have been obtained for [^11^C]glutamate and [^11^C]aspartate, also in rats [21]. Moreover, in humans, not only did [^11^C]aspartate exhibit 2-fold lower kidney uptake compared with [^11^C]glutamate, but it also was excreted at a faster rate [21]. Therefore, the lower kidney uptake and concurrent higher accumulation in liver, spleen, and lungs of [^125^I]IB-Mal-d-GDDDK-5F7 compared to [^131^I]IB-Mal-d-GEEEK-5F7 (or more likely, labeled catabolites thereof) is in good agreement with results observed with [^11/14^C]aspartate and [^11/14^C]glutamate [21,33]. However, it is important to point out that in the present study, prosthetic groups built up of five amino acids were coupled to an sdAb molecule and were evaluated in contrast to a single labeled l-amino acid. Additionally, these prosthetic agents were composed of d-amino acids, a feature that can reduce proteolytic cleavage but also increase kidney uptake of labeled catabolites [8]. Finally, it is worth noting that the effects of glutamic acid residue content on the kidney uptake of renally filtered biomolecules are difficult to predict, because introduction of this negatively charged amino acid has been reported to both significantly reduce [41] and increase [42,43] activity levels in the kidney.

We recently developed another residualizing agent, [^*^I]IB-NHS-d-EEEG, which is an analogue of [^*^I]IB-Mal-d-GEEEK, in which the maleimide (Mal) functionality was replaced with an *N*-hydroxysuccinimide (NHS) ester to facilitate labeling proteins without a cysteine tag. Studies comparing the biodistribution of trastuzumab labeled using the two reagents demonstrated a 3- and 8-fold lower kidney uptake at 4 and 144 h, respectively, with [^*^I]IB-NHS-d-EEEG labeling [22], suggesting that the nature of the conjugating moiety can also influence biodistribution. Indeed, proteins radiolabeled by maleimide conjugation can have considerably higher kidney uptake of activity than when labeled by conjugation with an isothiocyanato- or *N*-hydroxysuccinimide-functionalized reagents [44]. Therefore, it might be worth synthesizing an [^*^I]IB-Mal-d-GDDDK analogue with an NHS ester functionality and evaluating the effect of this modification on the biodistribution of sdAbs labeled using this reagent. However, it is important to note that trastuzumab radioiodinated using [^*^I]IB-NHS-d-EEEG also exhibited lower tumor uptake [22]. This is an important factor to consider, given that tumor uptake of radiolabeled sdAbs is generally lower than that of radiolabeled intact mAbs. Nonetheless, given that both [^131^I]IB-Mal-d-GEEEK and [^125^I]IB-Mal-d-GDDDK contained a maleimide functionality, the kidney uptake differences observed in the current study most likely reflect substitution of glutamate by aspartate. Extensive analyses of labeled catabolites generated in vivo from proteins with molecular weights above and below the cut-off for renal filtration and labeled using various peptide-based residualizing agents are needed to have a better understanding of the mechanisms governing differences in biodistribution in general, and kidney uptake in particular. Such investigations could facilitate the development of a residualizing agent that allows maximal tumor uptake without excessive kidney retention. Finally, it is worth noting that kidney reduction strategies could also be applied, for example, coinjection of Gelofusine, an approach which has shown promise in combination with radiolabeled sdAbs [34,35].

## 3. Materials and Methods

### 3.1. General

All chemicals were purchased from Sigma-Aldrich (St. Louis, MO, USA), unless otherwise specified. Sodium [^125^I]iodide and sodium [^131^I]iodide in 0.1 M NaOH with molar activities of 81.4 TBq/mmol and 44.4 TBq/mmol, respectively, were purchased from Perkin-Elmer Life and Analytical Sciences (Boston, MA, USA). The d-YDDDD and d-YEEEE peptides were purchased from ProImmune Inc. (Bradenton, FL, USA). Unlabeled IB-Mal-d-GEEEK, its tin precursor *N*^ε^-(3-(tri-*n*-butylstannyl)benzoyl)-Lys^5^-*N*^α^-maleimido-Gly^1^-d-GEEEK (TB-Mal-d-GEEEK), and radioiodinated [^*^I]IB-Mal-d-GEEEK were synthesized as reported before [14]. *N*^α^-Fmoc-*N*^ε^-Boc-d-Lys attached to Wang resin and Fmoc-d-Asp-(O-*t*Bu)-OH) were obtained from Novabiochem (West Chester, PA, USA). Maleimido glycine (maleimido acetic acid) was synthesized following a literature procedure [45]. *N*-succinimidyl 3-iodobenzoate (SIB) and *N*-succinimidyl 3-(tri-*n*-butyl)stannyl-benzoate (STB) were prepared as described previously [46].

Solid-phase peptide synthesis was carried out on a Thermo-Fisher Applied Biosystems (Foster City, CA, USA) Model 433A automated peptide synthesizer employing Fast-Moc chemistry with HBTU activation of carboxyl groups. Mass spectra were recorded using an Agilent 1100 LC/MSD trap and an electrospray ionization (ESI) LC-MS spectrometer (Santa Clara, CA, USA). High-performance liquid chromatography (HPLC) was done using the following systems: (1) For analytical and semi-preparative HPLC of nonradioactive compounds, a Waters Model Delta 600 system with a Model 2487 dual wavelength absorbance detector was used; data were acquired using Millennium software; (2) For radiochemical HPLC separation, a Beckman Gold HPLC system equipped with a Model 126 programmable solvent module coupled with a Model 166 NM variable wavelength detector and a Model 170 radioisotope detector were used; data were acquired using 32 Karat software; (3) For size-exclusion HPLC, a Beckman Gold HPLC system with Model 168 diode array detector and an IN/US γ-RAM activity detector with Laura Lite software (Tampa, FL, USA) were used; this system can detect two radioisotopes simultaneously. A Waters XTerra C18 HPLC column (4.6 mm × 250 mm, 5 μm), eluted at a flow rate of 1 mL/min, was used for analytical and radioanalytical chromatography, and a Waters XTerra C18 column (19 mm × 150 mm, 7 μm), eluted at 6 mL/min, was used for semi-preparative chromatography. The gradient elution system utilized deionized water (A) and acetonitrile (B), each containing 0.1% (*v*/*v*) acetic acid. Two binary gradients were employed: Gradient 1—the proportion of solvent B was held at 5% for 10 min, linearly increased to 100% over next 30 min, held at 100% for 5 min, and finally brought back to initial conditions over next 5 min; and Gradient 2—the proportion of B was linearly increased from 50% to 100% over 30 min, held at this composition for 5 min and brought back to initial conditions over the next 5 min. Size-exclusion HPLC was performed using a TSK-GEL G3000SW column (7.5 mm × 600 mm; 10 μm; TOSOH Bioscience LLC, Montgomeryville, PA, USA), which was eluted with PBS (pH 7.4) at a flow rate of 0.2 mL/min.

### 3.2. Single Domain Antibody Fragment, Cells, and Culture Conditions

The version of the anti-HER2 sdAb 5F7 bearing a C-terminus GGC tail (hereafter referred to as 5F7) was a gift from Dr. Hilde Revets, formerly of Ablynx NV (Ghent, Belgium). This anti-HER2 sdAb was selected from phage libraries derived from *llamas* immunized with SKBR-3 human breast carcinoma cells, and details for production, purification, and characterization have been reported previously [17].

All reagents used in cell culture studies were purchased from Invitrogen (Grand Island, NY, USA). HER2-expressing BT474M1 human breast carcinoma cells [47], a more tumorigenic version of the original BT474 line, were grown in DMEM/F12 medium supplemented with 10% fetal bovine serum, streptomycin (100 μg/mL), and penicillin (100 IU/mL). HER2-expressing SKOV-3 human ovarian carcinoma cells, obtained from the Duke University Cell Culture Facility, were grown in McCoy’s 5A medium containing 10% fetal bovine serum and 1% penicillin-streptomycin. Cells were cultured at 37 °C in a 5% CO_2_ humidified incubator, medium was changed every two days, and cells were passaged by trypsinization (0.05% Trypsin-EDTA) when they were about 80% confluent. Trastuzumab (Herceptin^®^, Genentech, San Francisco, CA, USA) was obtained from the Duke Cancer Institute Pharmacy.

### 3.3. Synthesis

#### 3.3.1. Mal-d-GDDDK (**1**)

Mal-d-GDDDK was synthesized following the procedure used for Mal-d-GEEEK [14] by substituting glutamic acid residues with aspartic acid. Briefly, three Fmoc-d-Asp(O*t*Bu)-OH moieties and maleimido glycine were sequentially attached to *N*^α^-Fmoc-*N*^ε^-Boc-d-Lys anchored to Wang resin (0.1 mmol) in the automated synthesizer using the manufacturer’s Fast-Moc chemistry. Cleavage of the peptide from the resin and simultaneous removal of side-chain protecting groups were achieved by treatment with a 100:95:2.5:2.5 (*v*/*v*/*v*/*v*) cocktail of DCM:TFA:water:triisopropylsilane (TIS). The resin was filtered and washed three times with the cocktail, and the combined filtrate was concentrated to a small volume with a stream of argon. Cold diethyl ether was added to obtain a white solid. Analytical HPLC (Gradient 1): *t*_R_ = 4.9 min. LCMS (*m*/*z*) Calcd. for C_24_H_32_N_6_O_14_: 628.2. Found: 629.3 (M + H)^+^.

#### 3.3.2. TB-Mal-d-GDDDK (**2**)

A solution of Mal-d-GDDDK (11.6 mg; 18.4 μmol) in 350 μL of 1% (*v*/*v*) triethylamine in DMF was added to STB (43.7 mg; 86 μmol) in a ½-dram vial, purged with argon, and the mixture was stirred at room temperature for 48 h. Cold ether was added, and the resultant precipitate was pelleted, washed several times with cold ether, dissolved in 1:1 (*v*/*v*) water:acetonitrile, and purified by semi-preparative HPLC using Gradient 2 to obtain 5.1 mg (27%) of TB-Mal-d-GDDDK as a white powder. Analytical HPLC (Gradient 1): *t*_R_ = 37.6 min. LCMS (*m*/*z*) Calcd. for C_43_H_62_N_6_O_15_Sn: 1022.3. Found: 1023.4 (M + H)^+^, 1045.3 (M + Na)^+^.

#### 3.3.3. IB-Mal-d-GDDDK (**3**)

Mal-d-GDDDK (5.5 mg; 8.8 μmol) and SIB (16.6 mg; 48.1 μmol) were dissolved in 150 μL of 1% (*v*/*v*) triethylamine in DMF, and the homogenous mixture was stirred at room temperature overnight under an argon atmosphere. The product was precipitated by the addition of cold ether, pelleted by centrifugation, washed several times with cold ether, and dried to obtain 4.2 mg (55.9%) of a yellowish solid. Analytical HPLC using modified Gradient 1 (duration of the initial gradient step shortened to 5 min): *t*_R_ = 24.6 min. LCMS (*m*/*z*) Calcd. for C_31_H_35_IN_6_O_15_: 858.1. Found: 858.7 (M + H)^+^.

### 3.4. Radiochemistry

The d-YDDDD and d-YEEEE peptides were radioiodinated on their constituent tyrosine as reported [8]. Briefly, Na[^125^I]iodide or Na[^131^I]iodide (37 MBq) was added to d-YDDDD or d-YEEEE (10 μL, 1 mg/mL PBS) diluted with PBS (60 μL) in a glass vial coated with Iodogen (100 μg). The vial was placed on an orbital shaker for 40 min, and the mixture was diluted with 3 mL of water. The solution was passed through a tC18 cartridge (Waters, Milford, MA, USA), which was then washed with an additional 3 mL of water. Each radioiodinated d-peptide was eluted in 400 μL of acetonitrile containing 1% acetic acid. The solvent was evaporated to dryness under a stream of nitrogen, and the peptide residue was dissolved in the volume of PBS required for the biodistribution study.

Radioiodination of the TB-Mal-d-GDDDK tin precursor was performed similarly to the procedure reported for TB-Mal-d-GEEEK [14,17]. Briefly, 40 μL of glacial acetic acid (AcOH) was added to a ½-dram vial containing dried sodium [^125^I]iodide (40.7–196.1 MBq) followed by TB-Mal-d-GDDDK (20 μg/25 μL AcOH) and *N*-chlorosuccinimide (0.4 mg/20 μL AcOH). This mixture was incubated at room temperature for 15 min, and the labeled peptide was isolated by reversed-phase analytical HPLC using Gradient 1. The fractions containing the major radioactive peak and having a retention time corresponding to **3** (*t*_R_ = 29.5 min) were collected, purged with an argon stream to remove most of the acetonitrile, diluted with water, and concentrated by solid-phase extraction using a Sep-Pak classic C18 cartridge (Waters, Milford, MA, USA). The radioiodinated peptide was eluted from the cartridge with 1 mL of anhydrous methanol, the solvent was evaporated from the eluate with a stream of argon, and the residual activity was used as such for reaction with the sdAb.

### 3.5. Labeling of sdAb

Coupling of the [^125^I]IB-Mal-d-GDDDK and [^131^I]IB-Mal-d-GEEEK prosthetic groups to the sdAb was performed using a previously described procedure [17]. Briefly, 100 µL of thiolated 5F7 (89 µg, 6.8 nmol) in 0.1 M phosphate buffer (PB), pH 7.0 with 5 mM EDTA was added to a ½-dram vial containing the dried prosthetic agent (7.4–111 MBq). After vortexing the vial, the mixture was incubated at room temperature for 45 min. The reaction was quenched by incubation with iodoacetamide (10 μL; 100 mg/mL in PB/EDTA pH 7.0) for an additional 15 min. Finally, the labeled sdAb was isolated by gel filtration on a PD-10 column (GE Healthcare, Piscataway, NJ, USA) eluted with PBS pH 7.4, and collected in 250 µL fractions. For determination of *K*_D_, 5F7 sdAb was labeled using [^125^I]IB-Mal-d-GEEEK using the same procedure.

### 3.6. Evaluation of Protein-Associated Activity and Immunoreactivity

Protein-associated activity in the radioiodinated sdAb preparations was determined in a single or, when appropriate, paired-label format, using three assays [17,18]. ITLC was performed by developing silica gel-impregnated glass fiber sheets (Pall Corporation, East Hill, NY, USA) with PBS, pH 7.4. Under these conditions, the labeled sdAb remained at the origin, and small molecular weight components moved with an R_f_ of 0.6–0.8. The sheets were cut into small strips and counted for activity. TCA precipitability was determined by incubating about 5 ng of each labeled sdAb with 800 μL of 2% bovine serum albumin (BSA) and 100 μL of 20% trichloroacetic acid (TCA) at room temperature for 15 min. The mixtures were pelleted, and both pellets and supernatants counted; protein-associated activity was calculated as the percent of total activity present in the pellet. Selected batches of radiolabeled sdAbs also were analyzed by SDS-PAGE under non-reducing conditions using Mini-PROTEAN TGX Any kD gels (BioRad, Hercules, CA, USA) and subsequently visualized using a Storage Phosphor Cyclone Plus System (Perkin-Elmer Life and Analytical Sciences, Downers Grove, IL, USA) as previously described [17]. Size-exclusion HPLC also was performed as reported before [15].

Immunoreactivity was determined by incubating the radiolabeled sdAb preparations for 30 min at 37 °C with a 10–20-fold molar excess of recombinant HER2 extracellular domain (HER2-ECD) coupled to the Fc region of human IgG (131 kDa) (R&D Systems, Minneapolis, MN, USA) in 0.1 M PBS pH 7.4. The fraction of activity bound to HER2-ECD was determined by injecting this mixture onto a size-exclusion HPLC column [15]. The immunoreactive fraction (IRF) of the labeled sdAbs also was determined using a modified Lindmo assay that utilized magnetic beads coated with either recombinant HER2-ECD (positive) or BSA (negative control) as described [17,18]. Triplicate samples were used for each concentration, and the entire experiment was repeated six times.

### 3.7. Determination of Binding Affinity (K_D_)

The binding affinity of [^125^I]IB-Mal-d-GDDDK-5F7 and [^125^I]IB-Mal-d-GEEEK-5F7 for HER2 was determined using a saturation binding assay on the HER2-expressing BT474M1 cell line. Cells were seeded in 24-well plates at a density of 8 × 10^4^ cells/well and incubated at 37 °C for 24 h. On the next day, the cells were incubated in triplicate with increasing concentrations (0.1–100 nM; final volume 600 μL) of [^125^I]IB-Mal-d-GDDDK-5F7 or [^125^I]IB-Mal-d-GEEEK-5F7 for 2 h at 4 °C. A parallel assay was performed in which cells were coincubated with a 100-fold molar excess of trastuzumab, which competes with 5F7 for HER2 binding [17], to determine nonspecific binding at each concentration. The medium containing unbound activity was removed, and cells were washed twice with cold PBS and solubilized by treatment with 1 M NaOH (0.5 mL) at 37 °C for 10 min. Cell-associated activity was determined using a Perkin Elmer Wizard II (Shelton, CT, USA) dual-channel gamma counter. The dissociation constant (*K*_D_) was determined by nonlinear regression using GraphPad Prism software (version 5.01). Triplicate samples were used for each concentration, and the entire experiment was repeated twice.

### 3.8. Paired-Label Internalization Assay

BT474M1 cells at density of 8 × 10^5^ cells/well were plated in 6-well plates, incubated at 37 °C overnight, brought to 4 °C, and incubated for 30 min. Media was replaced with fresh media containing 5 nM each of [^125^I]IB-Mal-d-GDDDK-5F7 and [^131^I]IB-Mal-d-GEEEK-5F7, and the cells were incubated at 4 °C for 1 h. Next, medium containing unbound activity was removed, cells were washed with cold PBS, fresh medium was added, and the cells incubated at 37 °C for various time points up to 24 h. Parallel experiments were performed with a 100-fold molar excess of trastuzumab to determine nonspecific uptake. The percentage of initially cell bound activity that was internalized, membrane-bound, and that had leaked into cell culture supernatants over the 24-h assay period was determined. Protein-associated activity in the cell culture supernatants was determined by TCA precipitation. Triplicate samples were used for each time point, and the entire experiment was repeated twice.

### 3.9. Paired-Label Biodistribution

Animal studies were performed under the guidelines established by the Duke University Institutional Animal Care and Use Committee. An initial paired-label biodistribution study with the labeled d-peptides was performed in Balb/c mice weighing 20–25 g. Groups of five animals were injected intravenously with 148 kBq (40 ng) each of d-[^125^I]YDDDD and d-[^131^I]YEEEE and then killed by isofluorane overdose 0.5, 1, 2, and 24 h later. A second biodistribution study was performed to compare 5F7 sdAb labeled with [^125^I]IB-Mal-d-GDDDK and [^131^I]IB-Mal-d-GEEEK in Balb/c mice to assess the kidney retention of both radioconjugates. Groups of five mice received 129.5 kBq each of [^125^I]IB-Mal-d-GDDDK-5F7 (1.4 μg, 92.5 kBq/μg) and [^131^I]IB-Mal-d-GEEEK-5F7 (0.5 μg, 259 kBq/μg) via the lateral tail vein in a total volume of 100 μL PBS. At 1, 2, 4, 8, and 24 h post injection, mice were euthanized by isofluorane overdose, dissected, and organs isolated. The third paired-label biodistribution study was performed in athymic mice bearing subcutaneous SKOV-3 tumor xenografts. These xenografts were established as described previously [48], and biodistribution was evaluated when tumors reached a volume of about 350–500 mm^3^. Groups of four mice received intravenous injections of [^125^I]IB-Mal-d-GDDDK-5F7 (155.4 kBq, 0.3 μg, 518 kBq/μg) and [^131^I]IB-Mal-d-GEEEK-5F7 (162.8 kBq, 0.4 μg, 407 kBq/μg). At 1, 4, and 24 h, mice were euthanized by isofluorane overdose, dissected, and blot-dried tissues were weighed and counted along with injection standards in the dual-channel gamma counter. Results were expressed as percentage of injected dose per gram of tissue (% ID/g), except for thyroid, for which % ID/organ values were calculated.

Data are presented as mean ± standard deviation. The statistical significance of differences in uptake between the two tracers was determined via a paired two-tailed Student *t*-test using Microsoft Excel. A *p* value less than 0.05 was considered statistically significant.

## 4. Conclusions

[^*^I]IB-Mal-d-GDDDK was synthesized and compared with [^*^I]IB-Mal-d-GEEEK, a residualizing prosthetic agent that had previously been demonstrated to offer clear advantages for labeling internalizing mAbs including excellent retention in tumor, minimal dehalogenation, and a low degree of uptake in normal tissues. Simple substitution of d-glutamates for d-aspartates in [^*^I]IB-Mal-d-GEEEK did not compromise HER2 targeting in vitro and in vivo. Radioiodination of 5F7 using [^*^I]IB-Mal-d-GDDDK was achieved in radiochemical yields similar to those obtained with [^*^I]IB-Mal-d-GEEEK. Paired-label internalization assays indicated that both residualizing labels were equivalent with respect to intracellularly trapping of activity in BT474M1 cells in vitro. Biodistribution studies in mice with SKOV-3 xenografts indicated that the major difference between the two residualizing labels was in their kidney uptake. Future experiments will be performed to evaluate the nature of the labeled catabolites generated from proteins labeled using both residualizing labels to better understand the factors responsible for their kidney uptake.

## Figures and Tables

**Table 1:** Paired-label biodistribution of 5F7 sdAb labeled using [^125^I]IB-Mal-d-GDDDK and [^131^I]IB-Mal-d-GEEEK in athymic mice with subcutaneous SKOV-3 human ovarian carcinoma xenografts.

Organ/Tissue	%ID/g ^1^					
	1 h		4 h		24 h	
	I-125	I-131	I-125	I-131	I-125	I-131
Liver	4.15 ± 1.12	1.99 ± 0.50	4.59 ± 0.53	1.73 ± 0.09	2.52 ± 0.71	0.56 ± 0.17
Spleen	2.28 ± 1.00	1.05 ± 0.34	1.67 ± 0.69	0.73 ± 0.17	1.68 ± 0.55	0.36 ± 0.12
Lungs	10.34 ± 2.37	4.81 ± 0.93	7.00 ± 2.13	1.86 ± 0.29	2.30 ± 0.68	0.52 ± 0.35
Heart	1.34 ± 0.37	0.99 ± 0.34	0.67 ± 0.12	0.35 ± 0.01	0.25 ± <0.01	0.05 ± 0.01
Kidneys	127.5 ± 18.7	271.4 ± 66.6	132.2 ± 31.1	201.1 ± 41.1	94.6 ± 22.9 ^2^	81.4 ± 28.8
Stomach	0.80 ± 0.46 ^2^	0.83 ± 0.49	0.79 ± 0.30 ^2^	0.80 ± 0.31	0.14 ± 0.08 ^2^	0.06 ± 0.04
Small intestine	1.20 ± 0.59 ^2^	1.18 ± 0.60	0.83 ± 0.05 ^2^	0.77 ± 0.06	0.13 ± 0.03	0.05 ± 0.01
Large intestine	0.47 ± 0.12 ^2^	0.43 ± 0.12	1.89 ± 1.14 ^2^	1.84 ± 1.13	0.33 ± 0.20	0.15 ± 0.11
Thyroid	0.06 ± 0.05 ^2^	0.07 ± 0.07	0.05 ± 0.02 ^2^	0.05 ± 0.02	0.08 ± 0.02 ^2^	0.09 ± 0.02
Muscle	0.82 ± 0.12	0.58 ± 0.05	0.26 ± 0.09 ^2^	0.22 ± 0.05	0.13 ± 0.02	0.03 ± 0.01
Blood	2.20 ± 0.65	2.00 ± 0.68	0.49 ± 0.14 ^2^	0.45 ± 0.22	0.07 ± 0.01	0.05 ± 0.01
Bone	0.68 ± 0.13 ^2^	0.64 ± 0.11	0.27 ± 0.04 ^2^	0.28 ± 0.09	0.17 ± 0.02 ^2^	0.10 ± 0.04
Tumor	4.36 ± 0.77 ^2^	4.82 ± 1.00	3.28 ± 1.03 ^2^	3.49 ± 0.87	1.91 ± 0.32 ^2^	1.88 ± 0.39

^1^ Values are mean % ID/g ± SD (*n* = 4) except in the case of thyroid, for which % ID/organ values are given; ^2^ no significant difference in uptake of the two tracers (*p* > 0.05).

## Supplementary Materials

The following are available online: Figure S1: non-reducing SDS-PAGE/phosphor image profiles of [^125^I]IB-Mal-d-GDDDK-5F7 and [^131^I]IB-Mal-d-GEEEK-5F7; Table S1: paired-label biodistribution of d-[^125^I]YDDDD and d-[^131^I]YEEEE in Balb/c mice; Table S2: paired-label biodistribution of 5F7 sdAb labeled with [^125^I]IB-Mal-d-GDDDK and [^131^I]IB-Mal-d-GEEEK in Balb/c mice.
