# Supplementary material for: d-Amino Acid Peptide Residualizing Agents for Protein Radioiodination: Effect of Aspartate for Glutamate Substitution
Source: Molecules. 2018 May 20;23(5):1223. doi: 10.3390/molecules23051223 (PMC6099567; doi:10.3390/molecules23051223)
Supplement: Supplementary file 1 [file molecules-23-01223-s001.pdf]

# D-amino acid peptide residualizing agents for protein radioiodination: effect of aspartate for glutamate substitution

**Marek Pruszynski [1,2,*], Choong Mo Kang [1,3], Eftychia Koumarianou [1,4], Ganesan Vaidyanathan [1], Michael R. Zalutsky [1,*]**

[1] Department of Radiology, Duke University Medical Center, Durham, NC 27710, USA; ganesan.v@duke.edu (G.V); zalut001@mc.duke.edu (M.R.Z)

[2] Present address: Institute of Nuclear Chemistry and Technology, Warsaw, Poland; m.pruszynski@ichtj.waw.pl

[3] Present address: Korea Institute of Radiological and Medical Sciences, Seoul, South Korea; kcm1580@naver.com

[4] Present address Laboratory for Translational and Molecular Imaging, Duke-National University of Singapore Medical School, Singapore; eftychia.koumarianou@duke-nus.edu.sg

* Correspondence: zalut001@mc.duke.edu; Tel.: +1-919-684-7708; m.pruszynski@ichtj.waw.pl

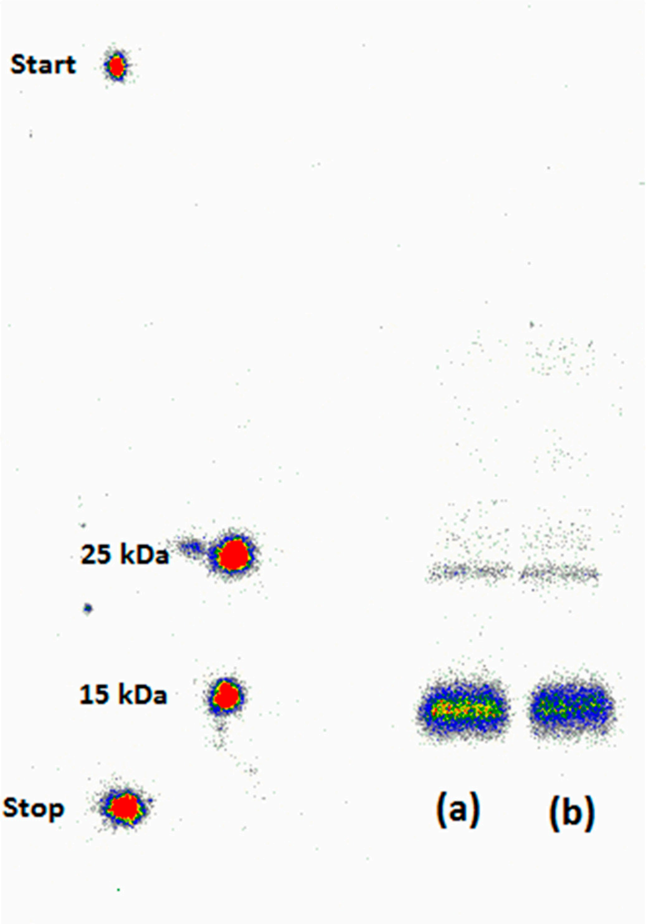


**Figure S1.** Non-reducing SDS-PAGE/phosphor image profiles of 5F7 sdAb: (**a**) [$^{125}$I]IB-Mal-D-GDDDK-5F7; (**b**) [$^{131}$I]IB-Mal-D-GEEEK-5F7. Two bands are visible with masses corresponding to about 12 kDa and 24 kDa, as this sdAb exists as a mixture of monomer and dimer as described previously [17].

**Table S1.** Paired-label biodistribution of D-[[125]I]YDDDD and D-[[131]I]YEEEE in Balb/c mice.

| Organ/Tissue | % ID/g [1] | | | | |
|---|---|---|---|---|---|
| | 0.5 h | 1 h | 2 h | 4 h | 24 h |
| ***D-[$^{125}$I]YDDDD*** | | | | | |
| Liver | 0.57 ± 0.05 | 0.36 ± 0.03 | 0.21 ± 0.02 | 0.19 ± 0.02 | 0.05 ± 0.01 |
| Spleen | 0.40 ± 0.04 | 0.18 ± 0.02 | 0.08 ± <0.01 | 0.09 ± 0.02 | 0.04 ± 0.01 |
| Lungs | 1.13 ± 0.11 | 0.47 ± 0.08 | 0.24 ± 0.09 | 0.09 ± 0.03 | 0.02 ± <0.01 |
| Heart | 0.47 ± 0.06 | 0.17 ± 0.03 | 0.06 ± 0.01 | 0.04 ± 0.01 | 0.01 ± <0.01 |
| Kidneys | 2.84 ± 0.52 | 1.20 ± 0.30 | 0.73 ± 0.07 | 0.57 ± 0.08 | 0.18 ± 0.05 |
| Stomach | 0.93 ± 0.14 | 0.67 ± 0.20 | 0.44 ± 0.15 | 0.34 ± 0.13 | 0.06 ± 0.03 |
| Small intestine | 0.47 ± 0.11 [2] | 0.30 ± 0.05 [2] | 0.14 ± 0.05 | 0.09 ± 0.03 | 0.06 ± 0.05 |
| Large intestine | 0.35 ± 0.15 [2] | 0.16 ± 0.04 | 0.16 ± 0.06 | 0.19 ± 0.01 [2] | 0.06 ± 0.02 [2] |
| Thyroid | 0.05 ± 0.01 | 0.05 ± 0.04 | 0.07 ± 0.03 [2] | 0.07 ± 0.03 | 0.11 ± 0.03 |
| Muscle | 0.62 ± 0.14 | 0.53 ± 0.19 | 0.20 ± 0.16 | 0.09 ± 0.06 [2] | 0.03 ± 0.02 [2] |
| Blood | 1.10 ± 0.22 | 0.31 ± 0.04 | 0.10 ± 0.02 | 0.06 ± 0.01 | <0.01 |
| Bone | 6.31 ± 1.10 | 4.85 ± 2.73 | 3.01 ± 0.61 | 1.97 ± 0.51 | 1.16 ± 0.33 |
| Brain | 0.06 ± 0.01 | 0.08 ± 0.06 | 0.02 ± 0.01 | 0.01 ± <0.01 | <0.01 |
| ***D-[$^{131}$I]YEEEE*** | | | | | |
| Liver | 0.44 ± 0.04 | 0.22 ± 0.02 | 0.11 ± 0.01 | 0.11 ± 0.01 | 0.02 ± <0.01 |
| Spleen | 0.33 ± 0.02 | 0.13 ± 0.02 | 0.06 ± <0.01 | 0.05 ± 0.02 | 0.02 ± <0.01 |
| Lungs | 0.92 ± 0.10 | 0.32 ± 0.05 | 0.18 ± 0.12 | 0.06 ± 0.03 | 0.02 ± <0.01 |
| Heart | 0.42 ± 0.06 | 0.12 ± 0.02 | 0.04 ± <0.01 | 0.03 ± 0.01 | <0.01 |
| Kidneys | 7.60 ± 1.40 | 5.39 ± 1.69 | 5.27 ± 0.48 | 4.43 ± 0.81 | 0.88 ± 0.09 |
| Stomach | 0.79 ± 0.13 | 0.54 ± 0.16 | 0.34 ± 0.11 | 0.27 ± 0.10 | 0.05 ± 0.03 |
| Small intestine | 0.47 ± 0.12 | 0.31 ± 0.06 | 0.13 ± 0.05 | 0.08 ± 0.03 | 0.05 ± 0.04 |
| Large intestine | 0.32 ± 0.12 | 0.14 ± 0.04 | 0.16 ± 0.05 | 0.19 ± 0.01 | 0.06 ± 0.02 |
| Thyroid | 0.03 ± 0.02 | 0.04 ± 0.02 | 0.05 ± 0.01 | 0.04 ± 0.02 | 0.08 ± 0.02 |
| Muscle | 0.44 ± 0.05 | 0.40 ± 0.21 | 0.07 ± 0.06 | 0.06 ± 0.03 | 0.01 ± <0.01 |
| Blood | 0.99 ± 0.20 | 0.22 ± 0.04 | 0.06 ± 0.01 | 0.04 ± 0.01 | 0.01 ± <0.01 |
| Bone | 1.83 ± 1.01 | 0.49 ± 0.23 | 0.25 ± 0.14 | 0.13 ± 0.02 | 0.07 ± 0.02 |
| Brain | 0.05 ± 0.01 | 0.06 ± 0.05 | 0.01 ± <0.01 | 0.01 ± <0.01 | <0.01 |

[1] Values are mean % ID/g ± SD (n = 4) except in the case of thyroid for which % ID/organ values given; [2] No significant difference in uptake of the two tracers (*P* >0.05).

**Table S2.** Paired-label biodistribution of 5F7 sdAb labeled with [$^{125}$I]IB-Mal-D-GDDDK and [$^{131}$I]IB-Mal-D-GEEEK in Balb/c mice.

| Organ/Tissue | %ID/g [1] | | | | |
|---|---|---|---|---|---|
| | 1h | 2h | 4h | 8h | 24h |
| ***[$^{125}$I]IB-Mal-D-GDDDK*** | | | | | |
| Liver | 3.34 ± 0.31 | 2.98 ± 0.20 | 2.84 ± 0.21 | 2.66 ± 0.25 | 1.65 ± 0.20 |
| Spleen | 2.35 ± 0.60 | 2.21 ± 0.16 | 2.38 ± 0.42 | 2.57 ± 0.25 | 2.21 ± 0.47 |
| Lungs | 6.34 ± 1.31 | 5.44 ± 1.43 | 4.02 ± 1.10 | 2.39 ± 1.00 | 1.40 ± 0.48 |
| Heart | 0.47 ± 0.06 | 0.27 ± 0.04 | 0.20 ± 0.04 | 0.12 ± 0.01 | 0.07 ± 0.01 [2] |
| Kidneys | 62.3 ± 13.7 | 54.9 ± 5.4 | 58.2 ± 5.2 | 34.1 ± 5.6 | 12.5 ± 2.4 |
| Stomach | 0.60 ± 0.27 [2] | 0.58 ± 0.20 [2] | 0.43 ± 0.15 [2] | 0.17 ± 0.03 [2] | 0.07 ± 0.03 [2] |
| Small intestine | 0.79 ± 0.13 | 0.94 ± 0.21 | 0.35 ± 0.08 | 0.17 ± 0.02 | 0.08 ± 0.02 [2] |
| Large intestine | 0.22 ± 0.07 | 0.24 ± 0.02 [2] | 0.62 ± 0.07 | 0.81 ± 0.12 | 0.12 ± 0.05 |
| Thyroid | 0.06 ± 0.06 | 0.05 ±<0.01 [2] | 0.03 ± 0.01 | 0.03 ±<0.01 [2] | 0.08 ± 0.03 [2] |
| Muscle | 0.30 ± 0.06 | 0.26 ± 0.10 | 0.10 ± 0.02 | 0.06 ± 0.01 [2] | 0.04 ± 0.01 [2] |
| Blood | 0.91 ± 0.09 | 0.73 ± 0.41 | 0.36 ± 0.18 | 0.15 ± 0.03 | 0.06 ± 0.01 |
| Bone | 0.82 ± 0.28 [2] | 0.60 ± 0.15 [2] | 0.34 ± 0.04 [2] | 0.27 ± 0.07 | 0.31 ± 0.10 |
| Brain | 0.05 ± 0.02 | 0.03 ± 0.01 | 0.01 ±<0.01 | 0.02 ± 0.01 [2] | <0.01 |
| ***[$^{131}$I]IB-Mal-D-GEEEK*** | | | | | |
| Liver | 1.80 ± 0.15 | 1.72 ± 0.31 | 1.37 ± 0.09 | 1.10 ± 0.12 | 0.54 ± 0.07 |
| Spleen | 1.29 ± 0.22 | 1.04 ± 0.08 | 0.87 ± 0.09 | 0.80 ± 0.14 | 0.43 ± 0.10 |
| Lungs | 1.78 ± 0.19 | 1.45 ± 0.10 | 0.77 ± 0.29 | 0.28 ± 0.03 | 0.17 ± 0.04 |
| Heart | 0.67 ± 0.12 | 0.40 ± 0.06 | 0.26 ± 0.05 | 0.14 ± 0.01 | 0.07 ± 0.02 |
| Kidneys | 128.6 ± 23.7 | 107.3 ± 10.3 | 106.3 ± 8.5 | 53.7 ± 7.2 | 20.3 ± 2.4 |
| Stomach | 0.60 ± 0.18 | 0.59 ± 0.21 | 0.42 ± 0.14 | 0.16 ± 0.02 | 0.07 ± 0.02 |
| Small intestine | 0.66 ± 0.10 | 0.71 ± 0.16 | 0.29 ± 0.08 | 0.13 ± 0.01 | 0.07 ± 0.01 |
| Large intestine | 0.26 ± 0.06 | 0.24 ± 0.03 | 0.45 ± 0.03 | 0.47 ± 0.11 | 0.09 ± 0.04 |
| Thyroid | 0.08 ± 0.05 | <0.01 | 0.05 ± 0.02 | 0.03 ± 0.01 | 0.07 ± 0.03 |
| Muscle | 0.35 ± 0.05 | 0.31 ± 0.08 | 0.13 ± 0.02 | 0.07 ± 0.02 | 0.04 ± 0.01 |
| Blood | 1.60 ± 0.18 | 1.17 ± 0.30 | 0.66 ± 0.24 | 0.30 ± 0.07 | 0.12 ± 0.01 |
| Bone | 0.75 ± 0.23 | 0.57 ± 0.12 | 0.28 ± 0.04 | 0.13 ± 0.05 | 0.16 ± 0.04 |
| Brain | 0.06 ± 0.01 | 0.05 ± 0.01 | 0.03 ±<0.01 | 0.01 ± 0.01 | 0.01 ±<0.01 |

[1] Values are mean % ID/g ± SD (n = 5) except in the case of thyroid for which % ID/organ values given; [2] No significant difference in uptake of the two tracers (*P* >0.05).
